# Draft Genome Sequence of *Arthrobacter chlorophenolicus* Strain Mor30.16, Isolated from the Bean Rhizosphere

**DOI:** 10.1128/genomeA.00360-15

**Published:** 2015-05-07

**Authors:** José Antonio Miranda-Ríos, José Augusto Ramírez-Trujillo, Bárbara Nova-Franco, Luis Fernando Lozano-Aguirre Beltrán, Gabriel Iturriaga, Ramón Suárez-Rodríguez

**Affiliations:** a Laboratorio de Fisiología Molecular de Plantas, Centro de Investigación en Biotecnología, Universidad Autónoma del Estado de Morelos, Cuernavaca, Morelos, México; b Programa de Genómica Evolutiva, Centro de Ciencias Genómicas, Universidad Nacional Autónoma de México, Cuernavaca, Morelos, México; c Tecnológico Nacional de México, Instituto Tecnológico de Roque, Roque Celaya, Guanajuato, México

## Abstract

Bacteria of the genus *Arthrobacter* are commonly found in the soil and plant rhizosphere*.* In this study we report the draft genome of *Arthrobacter chlorophenolicus* strain Mor30.16 that was isolated from rhizosphere of beans grown in Cuernavaca Morelos, Mexico. This strain promotes growth and ameliorates drought stress in bean plants.

## GENOME ANNOUNCEMENT

Members of the genus *Arthrobacter*, Gram-positive bacteria belonging to the class *Actinobacteria*, are widely distributed in the environment and have been isolated from the soil and plant rhizosphere (1, 2). Some species of this genus can degrade several xenobiotic compounds, such as 4-chlorophenol and 4-nitrophenol (3, 4), and have a high tolerance to desiccation, starvation, and other environmental stresses (5, 6). The genome sequence of Mor30.16 will help to elucidate the genes involved in trehalose accumulation, plant growth promotion, and the reduction of the harmful effects caused by drought in beans.

The genomic DNA was extracted using a Qiagen DNA isolation kit and sequenced by an Illumina HiSeq2000 platform at Beijing Genomics Institute Americas. Short paired-end reads of 90 bp in length were generated, comprising 3,891,127 reads. An assembly generated with SPAdes version 3.1.1 (7) resulted in 41 contigs with a *N*_50_ of 233,152 bp, a coverage of 80×, and a GC content of 66%. Automatic gene prediction and functional annotation were carried out by using the RAST server (8), and a total of 4,009 protein-coding sequences, 47 tRNAs, 6 rRNAs, and 53 ribosomal proteins were predicted. Average nucleotide identity analysis revealed that the draft genome is 93.5% identical to *Arthrobacter chlorophenolicus* strain A6, while the 16s rRNA gene shows an identity of 99.51%. Based on the results of the genome annotation we find that *A. chlorophenolicus* strain Mor30.16 displays three trehalose biosynthetic pathways. The first one is the trehalose-6 phosphate (T6P) synthase and T6P phosphatase route, which is the most widely distributed in nature and is encoded by the *otsA* and *otsB* genes. It involves the transfer of glucose from UDP-glucose to glucose-6-phosphate to yield trehalose-6-phosphate, which is subsequently converted to trehalose (9). The second pathway involves the conversion of maltooligosaccharides present in starch to trehalose, and is encoded by *treY* and *treZ*. Lastly, there are two *treS* genes in the Mor30.16 genome for the transformation of maltose to trehalose by trehalose synthase.

### Nucleotide sequence accession numbers.

This whole-genome shotgun project has been deposited at DDBJ/EMBL/GenBank under the accession number JZTW00000000. The version described in this paper is version JZTW01000000.
